# Epigenetic Changes Induced by Air Toxics: Formaldehyde Exposure Alters miRNA Expression Profiles in Human Lung Cells

**DOI:** 10.1289/ehp.1002614

**Published:** 2010-12-09

**Authors:** Julia E. Rager, Lisa Smeester, Ilona Jaspers, Kenneth G. Sexton, Rebecca C. Fry

**Affiliations:** 1 Department of Environmental Sciences and Engineering, Gillings School of Global Public Health and; 2 Center for Environmental Medicine, Asthma, and Lung Biology, School of Medicine, University of North Carolina–Chapel Hill, Chapel Hill, North Carolina, USA

**Keywords:** air pollution, environment, formaldehyde, gene regulation, human lung cells, miRNA, systems biology

## Abstract

**Background:**

Exposure to formaldehyde, a known air toxic, is associated with cancer and lung disease. Despite the adverse health effects of formaldehyde, the mechanisms underlying formaldehyde-induced disease remain largely unknown. Research has uncovered microRNAs (miRNAs) as key posttranscriptional regulators of gene expression that may influence cellular disease state. Although studies have compared different miRNA expression patterns between diseased and healthy tissue, this is the first study to examine perturbations in global miRNA levels resulting from formaldehyde exposure.

**Objectives:**

We investigated whether cellular miRNA expression profiles are modified by formaldehyde exposure to test the hypothesis that formaldehyde exposure disrupts miRNA expression levels within lung cells, representing a novel epigenetic mechanism through which formaldehyde may induce disease.

**Methods:**

Human lung epithelial cells were grown at air–liquid interface and exposed to gaseous formaldehyde at 1 ppm for 4 hr. Small RNAs and protein were collected and analyzed for miRNA expression using microarray analysis and for interleukin (IL-8) protein levels by enzyme-linked immunosorbent assay (ELISA).

**Results:**

Gaseous formaldehyde exposure altered the miRNA expression profiles in human lung cells. Specifically, 89 miRNAs were significantly down-regulated in formaldehyde-exposed samples versus controls. Functional and molecular network analysis of the predicted miRNA transcript targets revealed that formaldehyde exposure potentially alters signaling pathways associated with cancer, inflammatory response, and endocrine system regulation. IL-8 release increased in cells exposed to formaldehyde, and results were confirmed by real-time polymerase chain reaction.

**Conclusions:**

Formaldehyde alters miRNA patterns that regulate gene expression, potentially leading to the initiation of a variety of diseases.

Current indoor and outdoor air quality contributes significantly to global increases in morbidity and mortality (Brunekreef and Holgate 2002; Burnett et al. 2001; Smith and Mehta 2003). Epidemiological studies have shown that formaldehyde, a known air toxic, causes increased risk of childhood and adult asthma (Rumchev et al. 2002; Wieslander et al. 1997), acute respiratory tract illness (Tuthill 1984), nasopharyngeal cancer (Vaughan et al. 2000), and possibly leukemia (Zhang et al. 2010a). In animal studies, strong links have been made between formaldehyde exposure and nasal carcinoma (Kerns et al. 1983). Furthermore, the International Agency for Research on Cancer (IARC) has classified formaldehyde as a known human carcinogen (IARC 2006).

In outdoor environments, formaldehyde is present due to direct emissions from anthropogenic and biogenic sources, and it is also formed as a secondary chemical product through hydrocarbon atmospheric chemistry [World Health Organization (WHO) 2001]. Anthropogenic sources of formaldehyde include automobile exhaust, power plants, manufacturing facilities, and incinerators (U.S. Environmental Protection Agency 2007; WHO 2001). Ambient air has been estimated to contain formaldehyde at levels between 0.0008 and 0.02 ppm (WHO 2001). Higher formaldehyde exposure occurs within indoor environments, where humans inhale levels estimated between 0.02 and 0.3 ppm, depending on the presence of tobacco smoke (WHO 2001). The highest formaldehyde levels are found in certain occupational environments, such as industries related to resin, plastics, wood, paper, insulation, textile, and chemical productions, as well as medical institutions using disinfectants and embalming products. In these high-exposure cases, occupational workers are exposed to, on average, approximately 0.74 ppm (WHO 2001).

Because formaldehyde is highly reactive and water soluble, > 95% of inhaled formaldehyde is predicted to be absorbed within the human respiratory tract (Overton et al. 2001). Although most inhaled formaldehyde is absorbed in the nasal and upper airways (Overton et al. 2001), much remains uncertain about the dosimetry and mechanisms underlying pulmonary responses to formaldehyde (Thompson et al. 2008). As a result, it is important to study the effects of gas exposure to the lower respiratory region. Recently, Speit et al. (2010) established the effects of formaldehyde exposure on DNA-damage protection of human A549 alveolar epithelial cells.

Previous research has shown that altered gene expression patterns exist in nasal and lung cells exposed to formaldehyde (Kim et al. 2002; Li et al. 2007). These changes in gene transcript profiles, which likely translate to changes in protein levels, could arise from altered microRNA (miRNA) expression. miRNAs are an abundant class of regulatory molecules that have received scientific attention for their ability to alter mRNA abundance. miRNAs are noncoding single-stranded RNA molecules approximately 22 nucleotides in length (Bartel 2004). One of the more established functions of miRNAs is translational inhibition of target mRNA molecules, which occurs when miRNAs base pair with 3′-untranslated regions of mRNAs, causing decreases in protein production (Filipowicz et al. 2008). Once paired with mRNAs, miRNAs can destabilize mRNAs and target their degradation through deadenylation (Filipowicz et al. 2008; O’Hara et al. 2009). The study of the dysregulation and modification of miRNA abundance has revealed miRNAs’ important roles in many diseases, including heart failure, hematological malignancies, and neurodegenerative disease (Divakaran and Mann 2008; Fabbri et al. 2008; Hébert and De Strooper 2009). In addition, studies have shown that miRNA expression profiling in tumor cells can aid in classifying cancer type, cancer state, and cellular response to treatment (Calin and Croce 2006; Lu et al. 2005; Ma et al. 2007).

Mammalian miRNAs are estimated to regulate 30% of all protein-coding genes through posttranscriptional modification (Filipowicz et al. 2008). Because miRNAs play such a pivotal role in human cellular gene regulation, more research is needed to understand the effects of environmental exposures on miRNA levels. To our knowledge, only one other study has investigated the effects of environmental air pollution on cellular miRNA abundance (Jardim et al. 2009). The authors showed that diesel exhaust particles affected miRNA expression related to inflammatory response pathways and tumorigenesis.

In the present study, we hypothesized that formaldehyde exposure can disrupt miRNA levels within lung cells. We tested this hypothesis by exposing human lung epithelial cells to formaldehyde using a direct air–liquid interface that physically mimics the human respiratory tract. Using microarray analysis, we assessed the expression levels of > 500 known miRNAs. We demonstrated that formaldehyde significantly alters the expression profiles of 89 miRNAs that are predicted to target mRNAs associated with numerous biological pathways related to cancer, inflammatory response, and endocrine system regulation. Confirming one of the most dysregulated inflammation-associated pathways possibly influenced through altered miRNA levels, interleukin (IL)-8 showed significantly increased protein expression levels in formaldehyde-exposed cells. Taken together, this research suggests a novel epigenetic mechanism by which formaldehyde may induce disease.

## Materials and Methods

### Cell culture

Human A549 type II lung epithelial cells derived from a human lung adenocarcinoma were cultured according to standard protocol [ATCC (American Type Culture Collection), Manassas, VA]. Cells were grown in growth media containing F-12K plus 10% fetal bovine serum (FBS) plus 1% penicillin and streptomycin. Cells were plated onto 24-mm-diameter collagen-coated membranes with 0.4 μm pores (Trans-CLR; Costar, Cambridge, MA). Upon confluence, cells were cultured in phenol red-free F-12K nutrient mixture without FBS. Immediately before exposure, medium above each membrane was aspirated in order to create direct air–liquid interface culture conditions. The medium beneath each membrane remained to supply nourishment for cells throughout the exposure.

### Formaldehyde treatment

Gaseous formaldehyde was generated by heating 143 mg paraformaldehyde (Aldrich Chemical Co., Milwaukee, WI; lot no. 05910EI) in an air-flushed U-tube until the powder was completely vaporized within a dark unirradiated 120-m^3^ environmental chamber. The walls of the chamber are made of chemically nonreactive film, as described previously (Sexton et al. 2004). The chamber was naturally humidified from preflushing with HEPA-filtered ambient air during cloudy conditions. This resulted in a formaldehyde concentration of 1 ppm (1.2 mg/m^3^), which was then drawn through a cellular exposure chamber (Modular Incubator Chamber; Billups-Rothenberg, Inc., Del Mar, CA) at 1.0 L/min. The exposure chamber was positioned within an incubator where CO_2_ was added to the formaldehyde exposure source stream at 0.05 L/min and a small water dish provided proper humidification. Prepared lung cells were exposed to 1 ppm formaldehyde for 4 hr, and mock-treated control cells were exposed to humidified air under similar conditions. Experiments were carried out with six technical replicates for each exposure condition, generating a total of 12 samples. After 9 hr, cells were scraped and stored at −80°C in TRIzol reagent (Invitrogen Life Technologies, Carlsbad, CA), and basolateral supernatants were aspirated and stored at −80°C.

### Cytotoxicity analysis

To measure the cytotoxicity of formaldehyde, we measured the enzyme lactate dehydrogenase (LDH) within the supernatant of each sample using a coupled enzymatic assay (Takara), according to the supplier’s instructions (Takara Bio Inc., Otsu, Japan). LDH fold change (FC) was calculated as the mean LDH activity for formaldehyde-exposed samples divided by control values.

### Microarray processing

RNA molecules of at least 18 nucleotides in length were isolated using Qiagen’s miRNeasy Kit according to the manufacturer’s protocol (Qiagen, Valencia, CA). RNA was quantified with the NanoDrop 1000 spectrophotometer (Thermo Scientific, Waltham, MA), and its integrity was verified with a 2100 Bioanalyzer (Agilent Technologies, Santa Clara, CA). RNA was labeled and hybridized to the Agilent Human miRNA Microarray (version 1) (Agilent Technologies), which measures the expression levels of 534 human miRNAs. Three of the six total samples from each exposure condition, three formaldehyde-exposed and three mock-treated samples, were hybridized using 400 ng input RNA per sample. RNA labeling and hybridization were performed according to the manufacturer’s protocol, and microarray results were extracted using Agilent Feature Extraction software. Data were submitted to the Gene Expression Omnibus database (National Center for Biotechnology Information 2010) and are available under accession no. GSE22365 (Edgar et al. 2002).

### Microarray analysis

The resulting expression levels for each of the miRNAs measured by the microarrays were calculated and filtered for miRNAs expressed above a background level (background was set at 30, approximating the median signal per array). This resulted in a reduction of probe sets from 12,033 to 4,900 records. Differential miRNA expression was defined as a significant difference in miRNA expression levels between treated and untreated samples, where the following three statistical requirements were set: *a*) FC ≥ 1.5 or ≤ −1.5 (treated vs. untreated); *b*) *p* < 0.005; and *c*) false discovery rate (FDR) < 0.005. *p*-Values and FDRs were generated using the Comparative Marker Selection tool in GenePattern (Broad Institute 2010; Reich et al. 2006). Here, 2,000 permutation tests were carried out using the signal-to-noise ratio (SNR) analysis, and smoothed *p*-values were determined for each miRNA. SNR is defined by the equation SNR = (μ_A_ − μ_B_)/( σ_A_ + σ_B_), where μ represents average sample intensity and σ represents standard deviation (Golub et al. 1999). SNRs have been shown to provide one of the most accurate classification prediction methods (Cho and Ryu 2002). FDRs were calculated as the expected fraction of false positives among probe sets reported as significant using the Benjamini and Hochberg procedure (Benjamini and Hochberg 1995). Targets for the most differentially expressed miRNAs were identified using the miRDB database (miRDB 2010; Wang 2008; Wang and El Naqa 2008), where targets with a score of > 70 were investigated.

### Enriched biological functions and network analysis

We performed enriched biological functions and molecular network analyses using IPA (Ingenuity Pathways Analysis; Ingenuity Systems 2011), which provides a collection of gene to phenotype associations, molecular interactions, regulatory events, and chemical knowledge accumulated to develop a global molecular network. The lists of putative targets for each miRNA were overlaid onto this global molecular network, where protein networks significantly associated with the targets were algorithmically constructed based on connectivity. Associated enriched canonical pathways within these networks were also identified. Functional analysis was carried out to identify biological functions and disease signatures most significantly associated with the input targets. Statistical significance of each biological function or disease was calculated using Fisher’s exact test, which generated a *p*-value signifying the probability that each function or disease was associated with the miRNA targets by chance alone. Only enriched functions with *p*-values < 0.005 were assessed.

### Quantitative real-time polymerase chain reaction (RT-PCR) verification of miRNA expression

We also tested expression levels of the five most significantly modified miRNAs using quantitative RT-PCR (qRT-PCR). TaqMan MicroRNA Primer Assays for hsa-miR-33 (ID 002135), hsa-miR-450 (ID 2303), hsa-miR-330 (ID 000544), hsa-miR-181a (ID 000516), and hsa-miR-10b (ID 002218) were used in conjunction with the TaqMan Small RNA Assays PCR kit (Applied Biosystems, Carlsbad, CA). We used the MyCycler Thermal Cyler (Bio-Rad, Hercules, CA) for the reverse- transcription step, and the Lightcycler 480 (Roche, Indianapolis, IN) for the real-time step. The same three control and three formaldehyde-exposed samples from the microarray were used for qRT-PCR, which was performed in technical duplicate. Statistical significance was evaluated using a *t*-test.

### IL-8 measurement

We measured the protein abundance of the cytokine IL-8 using the basolateral supernatant from all 12 samples. An OptEIA human IL-8 enzyme-linked immunosorbent assay (ELISA; BD Biosciences, San Jose, CA) was performed and analyzed according to the manufacturer’s protocol. Experiments were carried out with 12 technical replicates for each exposure condition. To identify outliers of scanned absorbance readings, we performed the Grubbs’ test using GraphPad software (GraphPad Software 2011); outliers were identified as those with < 5% probability of occurring as an outlier by chance alone, based on a normal distribution (Grubbs 1969). IL-8 FC was calculated as mean IL-8 activity for formaldehyde-exposed samples divided by control values. Statistical significance of the treated versus untreated IL-8 levels was calculated using a *t*-test with Welch’s correction.

## Results

### Formaldehyde exposure modulates miRNAs in human lung cells

**Figure 1:**
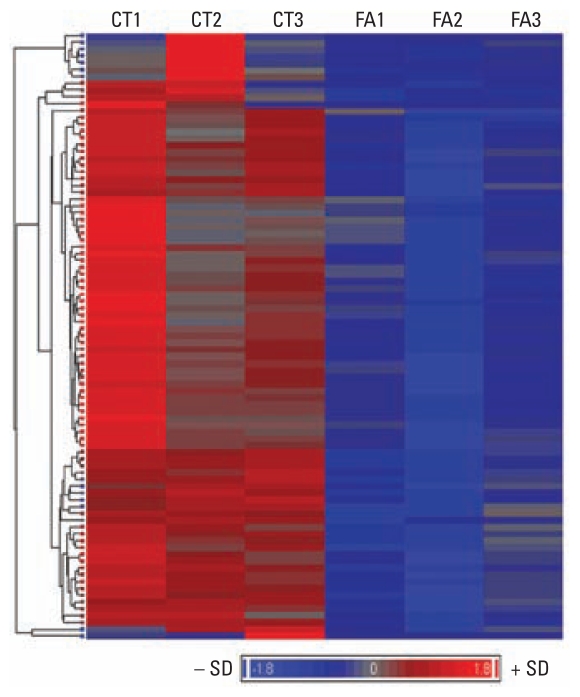
Heat map of 89 formaldehyde-modulated miRNAs. Data were mean standardized, and hierarchical clustering was performed. Blue indicates relative low expression, and red indicates relative high expression. Abbreviations: FA, formaldehyde-treated samples; CT, control samples.

In this study, we aimed to identify whether formaldehyde exposure alters the expression levels of miRNAs in lung cells. Human lung epithelial cells (A549) were exposed either to gaseous formaldehyde drawn directly from an unirradiated (dark) environmental chamber into an exposure chamber or were mock treated with humidified air as described. This exposure resulted in a 6.68-fold increase in LDH release associated with minimal cell killing in the formaldehyde-treated cells. After exposure, we collected small RNAs and measured their relative abundance using microarrays. A total of 343 unique miRNAs were detectable above background in these cells. The 343 miRNAs were further assessed for formaldehyde-induced changes in expression level. A total of 89 miRNAs showed a significant decrease in expression in the formaldehyde-exposed lung samples compared with control samples [Figure 1; see also Supplemental Material, Table 1 (doi:10.1289/ehp.1002614)]. We identified no miRNAs with significantly increased expression levels in response to formaldehyde. The five most significantly differentially expressed miRNAs, as determined through microarray analysis, were miR-33 (FC = −5.5), miR-450 (FC = −3.6), miR-330 (FC = −2.4), miR-181a (FC = −2.1), and miR-10b (FC = −2.1).

### miRNA expression changes validated through qRT-PCR

qRT-PCR validated the findings of the decreased miRNA expression induced by formaldehyde exposure: FC = −1.3 for miR-330; FC = −7.4 for miR-181a; FC = −1.2 for miR-33; and FC = −1.5 for miR-10b [see Supplemental Material, Figure 1 (doi:10.1289/ehp.1002614)]. miR-450 showed minimal expression changes (FC = −1.04; data not shown). Because we did not validate miR-450 results with qRT-PCR, no further analysis was performed on miR-450. To assess the similarity of the qRT-PCR and array-based quantification of the miRNA expression levels, we compared the average relative miRNA abundances with the raw microarray expression data (see Supplemental Material, Figure 1). This analysis shows high correlations (0.81 for control samples, 0.76 for treated samples) between the average miRNA abundance measured with both qRT-PCR and microarray (see Supplemental Material, Figure 1). More specifically, these analyses support that the direction of differential expression of the miRNAs induced by exposure to formaldehyde was consistent between the qRT-PCR and microarray analyses. It is important to note, however, that there is a difference in the magnitude of expression change, with the microarray results generally greater than those obtained with qRT-PCR (see Supplemental Material, Figure 1).

### miRNA targets are integrated into biological networks

In order to identify potential biological pathways affected by formaldehyde exposure, we ranked the 89 miRNAs that showed significant changes in expression levels according to their FCs, *p*-values, and qRT-PCR results [see Supplemental Material, Table 1 (doi:10.1289/ehp.1002614)]. Here, we further investigated the four miRNAs with the most significant formaldehyde-induced changes in expression:miR-33, miR-330, miR-181a, and miR-10b. For each of these four miRNAs, we identified their putative mRNA targets. Using a stringent cutoff of a match score between each miRNA and its mRNA targets followed by analysis for unique mRNAs per target list, we identified a total of 67 targets of miR-33, 217 targets of miR-330, 334 targets of miR-181a, and 25 targets of miR-10b (see Supplemental Material, Table 2). Among this list of 643 mRNAs, 42 were common to at least two of the modulated miRNAs (see Supplemental Material, Table 2).

**Figure 2:**
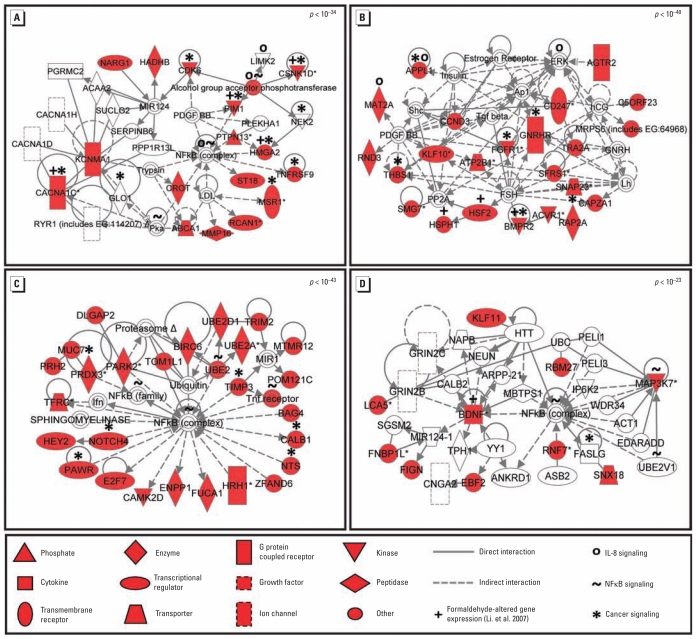
Significant molecular networks of modulated miRNAs affected by formaldehyde exposure. Protein networks display interactions using the mRNA targets of (*A*) miR-33, (*B*) miR-330, (*C*) miR-181a, and (*D*) miR-10b. *p*-Values representing the probability of these interactions occurring by chance are shown for each network (top right corner). Networks are displayed with symbols representing predicted miRNA targets (red symbols) or proteins associated with the predicted targets (open symbols).

Once we identified the predicted mRNA targets for the most significant miRNAs, we overlaid them onto molecular pathway maps enabled through the Ingenuity Pathways’ Knowledge Base (Ingenuity Systems, Redwood City, CA). Networks containing miRNA targets were algorithmically constructed based on connectivity and the known relationships among proteins. The predicted targets of miR-33, miR-330, miR-181a, and miR-10b generated a total of 40 networks [see Supplemental Material, Table 3 (doi:10.1289/ehp.1002614)]. For each of the miRNA gene targets, the most significant network (*p*-values ranged from 10^−23^ to 10^−43^) was highlighted for further evaluation (Figure 2). We queried the proteins identified within these networks for their enrichment for various canonical pathways. A comparison of the canonical pathways highlighted the conservation of a cancer-associated pathway common to all four miRNA-generated networks (see Supplemental Material, Table 4). Overlaying the pathway information onto the most significant networks resulted in the identification of enrichment for the nuclear factor κB (NFκB) pathway and the IL-8 signaling pathway, among others (Figure 2; see also Supplemental Material, Table 4).

Using a biological process enrichment analysis, we queried the 40 networks encoded by the mRNA targets for each miRNA to identify biological processes that were most significantly modulated by formaldehyde exposure; we found a total of 71 unique biological processes [see Supplemental Material, Table 5 (doi:10.1289/ehp.1002614)]. Across the mRNA targets, we found common enrichment for 13 different cellular biological processes. These processes included inflammatory response (*p* < 0.0029) and endocrine system development/function (*p* < 0.0018), which were enriched within the targets of all four miRNAs (Table 1).

### Conservation of predicted and observed mRNA targets

In our analysis, we used a stringent computational metric to match miRNAs to their predicted mRNA targets to better understand the biological implications of formaldehyde exposure. Because these mRNA targets were computationally predicted, we also compared our results with those of an existing genomic database established from a study that analyzed human tracheal fibroblast cells exposed to formaldehyde (Li et al. 2007). We found overlap between the predicted mRNA targets of the formaldehyde-modulated miRNAs and the tested formaldehyde-responsive genes previously identified (Li et al. 2007). Specifically, brain-derived neurotrophic factor (*BDNF*), bone morphogenetic protein receptor, type II (serine/threonine kinase) (*BMPR2*), calcium channel, voltage-dependent, L type, alpha 1C subunit (*CACNA1C*), casein kinase 1, delta (*CSNK1D*), high mobility group AT-hook 2 (*HMGA2*), heat shock transcription factor 2 (*HSF2*), heat shock 105kDa/110kDa protein 1 (*HSPH1*), and Pim-1 oncogene (*PIM1*) were present within the four most significant networks associated with the identified miRNA targets (Figure 2).

We expanded our comparison by performing network analysis on the formaldehyde-associated genes identified by Li et al.(2007). We constructed networks and identified related biological functions, as done with the miRNA predicted target network analysis. Networks related to cancer (*p* = 1.9 × 10^−19^), inflammation (*p* = 1.1 × 10^−8^), and endocrine system disorders (*p* = 3.15 × 10^−4^) were generated (see Supplemental Material, Table 6 (doi:10.1289/ehp.1002614)].

### Inflammatory cytokine IL-8 is released in response to formaldehyde

Based on our findings from the canonical pathway and biological process enrichment analyses that showed the IL-8 pathway as potentially dysregulated by miRNAs associated with formaldehyde exposure, we set out to confirm whether IL-8 protein levels may be influenced by such exposure. After cells were exposed to formaldehyde, we assessed IL-8 protein release. The investigation of the inflammatory response protein IL-8 showed that human lung cells activate an inflammatory response after exposure to formaldehyde. Specifically, we observed an average 16.9-fold increase (*p* < 0.05) in cytokine release in formaldehyde-exposed cells relative to control samples [see Supplemental Material, Figure 2 (doi:10.1289/ehp.1002614)].

## Discussion

In this study, we exposed A549 human lung epithelial cells to formaldehyde using an *in vitro* exposure system that physically replicates *in vivo* human lung gas exposures (Bakand et al. 2005). Although we recognize that A549 cells may not completely mimic normal lung cell response, there are several advantages to using these cells for air toxics studies. For example, when exposed to gases at an air–liquid interface, A549 cells secrete enough surfactant to mimic airway surface tension (Blank et al. 2006). As a result, A549 cells are routinely used to study the effects of environmental air exposures (Jaspers et al. 1997; Sexton et al. 2004; Speit et al. 2010). Moreover, they have been proposed as a standardized model to study the lung epithelium (Foster et al. 1998).

Our microarray analysis revealed that formaldehyde exposure resulted in the down-regulation of 89 miRNAs. All of the modulated miRNAs were down-regulated by formaldehyde exposure. This general trend of miRNA down-regulation has been observed in rat lung cells exposed to cigarette smoke (Izzotti et al. 2009), as well as in multiple tumor cell types, including lung cancer, breast cancer, and leukemia (Lu et al. 2005).

We focused a detailed analysis on the four most significantly down-regulated miRNAs, as determined through microarray analysis and qRT-PCR: miR-33, miR-330, miR-181a, and miR-10b. These miRNAs have been studied to some extent, and knowledge about their regulation and association to disease is growing. For example, miR-33 shows decreased expression levels in tissues from patients with lung carcinomas (Yanaihara et al. 2006). Also, miR-330 expression levels have been measured at significantly lower levels in human prostate cancer cells compared with nontumorigenic prostate cells (Lee et al. 2009). Furthermore, miR-330 has been suggested to act as a tumor suppressor by regulating apoptosis of cancer cells (Lee et al. 2009). In addition, miR-10b shows altered expression levels within breast cancer tissue and is one of the most consistently dysregulated miRNAs able to predict tumor classification (Iorio et al. 2005; Ma et al. 2007). These findings suggest that miR-33, miR-330, and miR-10b may influence cellular disease state specifically related to cancer.

Formaldehyde exposure also altered the expression level of miR-181a, which has known associations with leukemogenesis (Marcucci et al. 2009). The specific link between formaldehyde exposure and leukemia is currently debated, because numerous epidemiological studies show evidence both for possible association to this disease (Pinkerton et al. 2004; Zhang et al. 2010b) and against it (Bachand et al. 2010; Marsh and Youk 2004). However, in the present study we evaluated miRNA expression in lung cells, which likely differ from leukemia target cells in responses to formaldehyde exposure or exposure to formaldehyde’s metabolic products. Nevertheless, it is worth highlighting the observation of the dysregulation of miR-181a upon exposure to formaldehyde.

To expand our analysis, we used a systems biology approach to understand the potential biological implications of the miRNA expression changes induced by acute formaldehyde exposure. For this analysis, we used a stringent computational matching approach to identify predicted mRNA targets for miR-33, miR-330, miR-181a, and miR-10b. The identified mRNA targets were used to construct associated molecular networks and were analyzed for their known involvement in signaling pathways and biological functions. The identified networks showed enrichment for various canonical pathways, including NFκB and IL-8 signaling. Although very few predicted targets overlapped among the four miRNAs, we found proteins involved with cancer mechanisms, including that of the NFκB pathway, within the miRNA target networks. Importantly, NFκB has clear links to inflammation and cancer development (Karin and Greten 2005; Schmid and Birbach 2008). Also related to inflammation, IL-8–related signaling molecules were present in the miRNA target networks. Previous studies have shown IL-8 release in lungs cells representing inflammatory response after exposure to other air pollutants (Jaspers et al. 1997; Sexton et al. 2004). In addition, investigations have shown increased IL-8 levels in lungs of patients with diseases such as acute lung injury (McClintock et al. 2008), adult respiratory distress syndrome (Jorens et al. 1992), and asthma (Bloemen et al. 2007). Inflammation is a recognized formaldehyde-induced response, because formaldehyde is known to irritate the respiratory system (Tuthill 1984) and increase asthmatic response (Rumchev et al. 2002; Wieslander et al. 1997). Our findings suggest that the canonical pathways associated with formaldehyde-induced miRNA alterations may affect the regulation of biological pathways associated with various disease states, including cancer and inflammation.

To further verify our results, we compared the protein levels of cytokine IL-8 in formaldehyde-exposed cells versus mock-treated controls; IL-8 showed significantly increased protein expression levels in the formaldehyde-exposed cells. These results support our findings that IL-8 signaling is altered in lung cells exposed to formaldehyde. Interestingly, IL-8 levels are also increased in formaldehyde-exposed lung cells after presensitization to tumor necrosis factor-α (TNFα) (Persoz et al. 2010). TNFα is a proinflammatory mediator shown to have increased levels upon exposure to formaldehyde (Bianchi et al. 2004). Our network analyses suggest that cytokine signaling may be altered through changes in miRNA expression levels. Supporting this is a recent study showing that modifications to miRNAs may influence the expression of cytokines, including IL-6 and IL-8 (Jones et al. 2009). Future research will test whether the observed miRNA expression changes are directly associated with IL-8 signaling.

In an effort to gain further understanding of formaldehyde’s effects on gene expression, we compared our results with those of an existing genomics database (e.g., mRNA) from a study that evaluated human lung cells exposed to formaldehyde (Li et al. 2007). Using the predicted targets in our most significant miRNA networks, we found that the following genes overlap with the existing database: *BDNF*, *BMPR2*, *CACNA1C*, *CSNK1D*, *HMGA2*, *HSF2*, *HSPH1*, and *PIM1*. These genes have been shown to play a role in various diseases. For example, *a*) *BDNF* modulates neurogenesis after injury to the central nervous system (Ming and Song 2005); *b*) *CSNK1D* has been identified as up-regulated in breast cancer tissue (Abba et al. 2007); *c*) *HMGA2* is oncogenic in many cells, including lung carcinoma cells, and is regulated by the tumor-suppressive miRNA let-7 (Lee and Dutta 2007); and *d*) *PIM1* is found at increased levels within prostate cancer tissue (Dhanasekaran et al. 2001). Network analysis of all formaldehyde-responsive genes identified by Li et al. (2007) revealed significant associations with cancer, inflammation, and endocrine system regulation, which also overlap with our findings. These genes are therefore linked with formaldehyde-induced changes in miRNA abundance as well as mRNA alterations, and they are related to a diverse range of cellular responses, including tumorigenesis.

## Conclusions

Our study provides evidence of a potential mechanism that may underlie the cellular effects induced by formaldehyde, namely, the modification of miRNA expression. We have identified a set of 89 miRNAs that are dysregulated in human lung cells exposed to formaldehyde. Mapping the most significantly changed miRNAs to their predicted mRNA targets and their network interactomes within the cell revealed the association between formaldehyde exposure and inflammatory response pathways. We also validated our findings by *a*) performing qRT-PCR; *b*) integrating our predicted networks with known formaldehyde-induced mRNA expression changes; and *c*) examining protein expression changes of a key inflammatory response mediator, IL-8. Future research will investigate whether the expression levels of these miRNAs may serve as potential biomarkers of formaldehyde exposure in humans. Such biomarkers can be used to better monitor human exposure to environmental toxicants and relate them to health effects. Based on our findings, we believe that miRNAs likely play an important role in regulating formaldehyde-induced gene expression and may represent a possible link between exposure and disease.

## Figures and Tables

**Table 1:** Biological functions significantly associated with all predicted target sets of miR-33, miR-330, miR-181a, and miR-10b.

Enriched functions	Average *p*-value
Cellular development	0.0011
Small molecule biochemistry	0.0015
Nervous system development and function	0.0016
Cell-to-cell signaling and interaction	0.0017
Cell morphology	0.0017
Tissue development	0.0017
Cellular function and maintenance	0.0017
Cellular movement	0.0017
Endocrine system development and function	0.0018
Gene expression	0.0018
Cellular growth and proliferation	0.0021
Inflammatory response	0.0029
Hematological system development and function	0.0033
